# General Secure Information Exchange Protocol for a Multiuser MIMO Relay Channel

**DOI:** 10.3390/e21111054

**Published:** 2019-10-28

**Authors:** Qiao Liu, Hui Li, Yong Wang

**Affiliations:** School of Cyber Engineering, Xidian University, Xi’an 710126, China; qiaoliu@xidian.edu.cn (Q.L.); wangyong@mail.xidian.edu.cn (Y.W.)

**Keywords:** multiuser relay channel, private information exchange, public information exchange, physical layer security, secrecy sum-rates, MIMO

## Abstract

Secure information exchange occurs in many recently emerging cooperative-based networks, such as 5G networks (especially those with a Device to Device architecture), the Internet of Things, and vehicular ad hoc networks. However, the existing information exchange protocols only focus on either pairwise information exchange or group information exchange, and none of these protocols enable private and public information exchange to occur simultaneously. Thus, a general secure information exchange protocol for a multiuser channel is desirable. With this motivation, this paper investigates simultaneous private and public information exchange in a Multiple Inputs Multiple Outputs (MIMO) multiuser relay channel. In an aim to achieve this goal, signal alignment is chosen as the core technique. With the designed precoding matrix for each user, private information is aligned with its exchange partner, and public information forms a coding chain at the relay. With the aligned private signal and public coding chain, neither an untrusted relay nor external eavesdroppers can recover the original individual information. Performance analyses of the proposed protocol are conducted. First, we conduct transmission performance analyses from the perspective of time slot cost. Second, we conduct a security analysis for private information exchange and public information exchange. Third, we conduct secrecy sum-rate analysis for three attack scenarios: an untrusted relay attack only, an eavesdropper attack only, and both an untrusted relay and eavesdropper attack. The simulations are conducted to demonstrate that the proposed protocol can enable simultaneous private and public information exchange while resisting attacks by an undesired receiver, an untrusted relay, and external eavesdroppers.

## 1. Introduction

With the development of Information Technology (IT), the past half-century has witnessed the coming of a new age, namely, the Information Age. Almost everyone’s life has benefited tremendously from the evolution of wireless communication, and many novel techniques have optimized the performance of wireless networks. Among these techniques, cooperative communication is playing increasingly important roles in emerging wireless communication networks. So-called cooperative communication is a concept that enables wireless network users to communicate through one or multiple helper nodes, which are known as relays. With the help of cooperative relays, the system can obtain a better path loss gain, diversity gain, and multiplexing gain. These advantages have led to the increasing involvement of cooperation-based networks in the architecture of emerging mobile systems to an extent that is similar to that of Device-to-Device (D2D) networks [1] and heterogeneous networks (HetNet) [2,3].

Although the evolution of wireless communication techniques has evidently improved their transmission efficiency, there are still many hidden risks that hamper further development. Among those risks, the security problem is a constant and significant issue. Wireless communication is more vulnerable to attacks than wired communication. In a wireless network, a trade-off is sometimes necessary, and transmission resources are reduced to satisfy security requirements. In particular, with the increasing development of many new techniques, such as supercomputers and cloud computing, the traditional cryptography-based security strategy will face unprecedented challenges [4,5]. Thus, a brand new direction, such as physical layer security (PHY security), should be considered to secure wireless communication systems. In a physical layer security approach, key sharing is unnecessary, so key management can be avoided. Besides that, PHY security can obtain a good balance between security, efficiency, and complexity to achieve high transmission efficiency as well as information-theoretic security at a limited cost.

With this motivation, we aim to investigate physical layer secure information exchange in a common cooperative communication model, namely, a multiuser relay channel. In addition, massive MIMO has been stipulated to be one of the key techniques for 5G, and multiple antennas have been equipped in mobile devices as well as giant equipment. These factors all reflect the significant value of multiple-antenna techniques, in both theory and practice, for future wireless systems. Thus, we consider the MIMO system in this work.

PHY security in cooperative relaying was first considered in [6]. In [7], the security problems in a cooperative relaying system were divided into two parts depending on the relay adversarial model: (1) the untrusted relay model and (2) the trusted relay model.

In the trusted relay model, the relay facilitates a secure transmission for legitimate users. PHY security was studied for one-way relay in [8]. In [8], different relaying protocols, namely, Amplify-and-Forward (AF), Decode-and-Forward (DF), Compute-and-Forward (CF), and Noise-and-Forward (NF), were discussed in the context of security. The letter [9] proposed two cooperative beamforming schemes for the AF model: the secrecy rate maximization scheme and the null-space scheme. MIMO one-way relaying was investigated in [10] using a scenario in which the attacker has access to the global CSI. Another approach to PHY security in cooperative communication is the use of a supportive jammer. In [11], artificial noise signals were transmitted by supportive jammers to protect the transmission in a two-hop system. To maximize the secrecy capacity, the authors of [12] proposed a scheme in which the best channel condition relay is in charge of information relaying, while other relays are in charge of sending jamming signals. The work in [13] considered a more complex model in which multiple sources transmit signals to multiple destinations with multiple relays through a smart jamming algorithm.

In the untrusted relay model, the relay itself acts as an untrusted node that may attempt to illegitimately recover the information contained in messages from users. This is a common situation in the ad hoc network since many potential unfriendly devices exist in such networks, and some of them are eager to wiretap messages by providing fake assistance. In the context of security, relay selection is an effective way to prevent an active attack on the relays. However, it is difficult for the relay selection method to identify relays that will obey the transmission protocol but are curious about the information they are relaying. Thus, PHY security plays more roles in this type of model. Relay selection schemes in the context of security have been the focus of many studies [14,15,16]. The secrecy capacity was investigated using the untrusted relay model in the pioneer work of [6]. Following that, the work of [17] showed that a higher secrecy capacity could be achieved with a CF strategy. A method that combined jamming and relay selection was proposed in [18] to prevent untrusted relays in a two-hop cooperative network. An interesting scheme was proposed in [19] in which the receiver acted as a helper by sending jamming signals to the untrusted relay. The work of [20] extended the research on the signal antenna system to the MIMO system for untrusted relays.

The two-way trusted relay model is another system that has great potential for improving the overall network performance in terms of efficiency as well as security. Usually, a DF protocol is applied at the relay, and with the relay’s decoding, the information forms an integration at the relay (see [21,22]). The MIMO technique could provide more PHY redundancy for secure transmission. In [23], an analog network coding scheme and distributed beamforming scheme were applied to obtain secure transmission and prevent one eavesdropper. Another null-space precoding approach was proposed in [24], in which the secrecy sum-rates were optimized with different power constraints. The authors in [25] presented a PHY network coding design with secure precoding for two-way MIMO trusted relay channels.

A number of recent works have focused on secure transmission in a MIMO multiuser relay channel. In [26], multiple source nodes transmitted private information to multiple destination nodes with the help of multiple relays. The work in [27] investigated multiuser secure communications with the Base Station using direct links as well as multiple relays. In [28], secure transmission was obtained by trading off reliability for multiuser Single Input Multiple Output (SIMO) relay channels. Moreover, in [29], secure downlink broadcasting was obtained with the help of a regenerative relay in a physical layer security approach. All these studies have proposed effective physical layer security protocols for multiuser relay channels; however, they have only focused on enabling one-way information forwarding instead of information exchange. Using a two-way untrusted relay model, the authors in [30] proposed a beamforming method for secure information exchange. In particular, this paper considered the untrusted relay situation, which is the first for relay channels. Moreover, the concept is of great value because it utilizes the alignment of the transmitting signals to provide users with security. Furthermore, secure information exchange was extended to multi-hop in [31,32], in which security was the basis of PHY network coding.

Two types of secure information exchange exist in a multiuser relay channel on account of there being multiple users. On the one hand, users can exchange private information with other specified users. In this direction, several works have focused on signal processing methods for each information exchange pair [33,34,35]. However, none of them have addressed the security problem in a multiuser relay channel. The authors of [36] were the only ones to propose a physical layer security scheme that was based on interference alignment. On the other hand, users can also exchange public information with all other users. In other words, all users are broadcasting their information and receive information from all other users; thus, this type of exchange is also called group information exchange (see [37]) or full information exchange (see [38,39]).

Another consideration is that cryptography approaches are still the main methods for securing a multiuser relay channel. In private information exchange, each communication pair shares a secret key to encrypt its own information. This situation is identical to typical end-to-end encryption. Since the conditions differ for public information exchange, algorithms have been developed for group situations particularly. The authors in [40] proposed a broadcast encryption protocol that enables one sender to broadcast its information to all other users. However, this protocol cannot be used for private information exchange. Fast group key agreement is a development for public information exchange and was studied in [41], but the generated key can only be used for encrypting public information, which can be decrypted by all other users in the group.

The literature review reveals that practical protocols have been proposed for either private information exchange or public information exchange, and none of these approaches enable the simultaneous occurrence of the two types of information exchange. Thus, it is desirable to design a novel protocol that allows for the secure exchange private information as well as public information. With this motivation, we propose a protocol for private and public information exchange using a physical layer security method. The core technique of the protocol is signal alignment, which forms a summed signal for each private information exchange partner and a coding chain for all public information.

The contribution of this work can be summarized as follows:

- We propose a novel physical layer secure protocol that enables simultaneous private information exchange and public information exchange. With a designed precoding matrix for each user, the private information of one user is aligned with its exchange partner, and the public information forms a coding chain at the relay. For the relay, the summed signal and coding chain cannot be divided into separated individual information. To an external eavesdropper, each transmitted signal acts as interference for other signals. To the best of our knowledge, this proposed protocol is the first to enable simultaneous private information exchange and public information exchange.
- We conducted a performance analysis of the proposed protocol. First, a transmission performance analysis was conducted from the perspective of time slot cost. We compared the time slot cost of the proposed protocol with that of the well-known Time Division Multiple Access (TDMA) protocol and network layer network coding protocol. Second, a security analysis was conducted. We analyzed attacks from different nodes for the both the private exchange and public exchange. Finally, a secrecy sum-rate analysis was conducted under three scenarios: an untrusted relay attack only, an eavesdropper attack only, and both an untrusted relay and eavesdropper attack.
- We demonstrate the performance of the proposed protocol with simulated numerical results. First, we show the numerical difference in time slot cost between the proposed protocol and the well-known TDMA and network coding protocols. Second, we show the bit error ratio between the intended receive node and the untrusted relay and the external eavesdropper. Finally, we show the numerical results of the secrecy sum-rates under the three different attacks described in the previous item.

The rest of this paper is organized as follows. Section 2 introduces the basic transmission model and the security model. With the described system model, Section 3 proposes a novel physical layer secure protocol that enables simultaneous private and public information exchange for a MIMO multiuser relay channel. Section 4 describes the performance analysis of the proposed information exchange protocol, and the performance analysis results from multiple simulations are demonstrated in Section 5. Finally, Section 6 concludes this work and describes possible future works that build on this paper.

## 2. System Model

In this section, we introduce the basic system model and some preliminaries for the proposed protocol. The transmission model, the security model, and basic conditions and definitions are introduced sequentially.

### 2.1. Transmission Model

**Figure 1:**
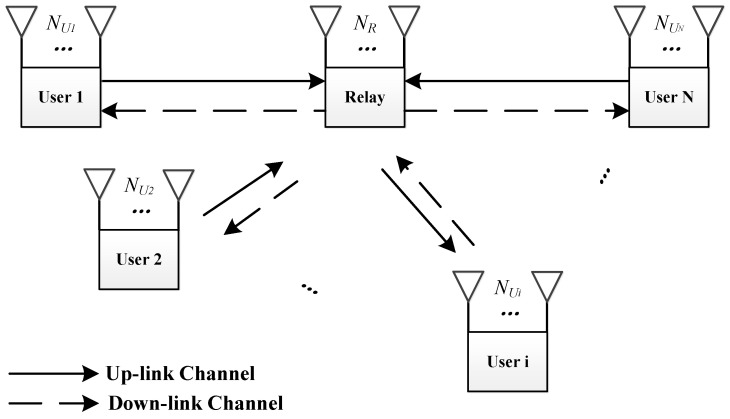
System model for a MIMO multiuser relay channel.

The basic model for the protocol is depicted in Figure 1. In this model, *N* users are willing to exchange information via one relay. In particular, we distinguish between information that is exchanged by a public message and a private message, and, to the best of our knowledge, this is a novel distinction. We discuss the details of these two types of messages in the section that addresses the security model.

The proposed protocol involves two time slots. In the first time slot, all *N* users send their signals simultaneously to the relay. After a simple operation, the relay broadcasts its signal to all *N* users. In our protocol, the relay does the physical layer network decoding first and then generates the signal to be broadcast in the second time slot. In other words, the proposed protocol is designed as a Decode-and-Forward (DF) model. Usually, the first time slot is called the uplink phase or multiple-access (MAC) phase, and the second time slot is called the downlink phase or broadcast (BC) phase.

If we consider the MIMO system, then all nodes in the system are equipped with multiple antennas. There are $N_{i}$ antennas equipped for user *i* and $N_{r}$ antennas equipped for the relay. We also assume that all the nodes work according to the half-duplex model.

We now move on to the formulation of this transmission model. In the MAC phase, each user applies physical layer network coding to its message. We use $m_{i}$ to denote the message to be transmitted for user *i*, and $c_{i}$ denotes the coded message. Then, we have $c_{i}=E\left(m_{i}\right)$, in which E denotes physical layer network coding. With the coded message, each user applies a precoding matrix $P_{i}$ to form the transmitted signal $X_{i}$ as

(1) $$X_{i}=P_{i}c_{i}.$$

In addition, the power constraint for the uplink phase is

(2) $$\sum_{i=1}^{N}\mathrm{Tr}\left(Q_{i}\right)\leq P_{T},$$

where $P_{T}$ is the total transmission power of all users, and $Q_{i}$ is the input covariances for user *i*. $Q_{i}$ is represented by $Q_{i}=E\left(X_{i}X_{i}^{t}\right)=E\left(p_{i}c_{i}c_{i}^{t}p_{i}^{t}\right)$, where $E\left(\cdot \right)$ is the expectation operator. Furthermore, the codewords are all independent, so $E\left(c_{i}c_{i}^{t}=I\right)$. Then, $Q_{i}$ is $Q_{i}=E\left(p_{i}p_{i}^{t}\right)$. Each user’s transmitted signal must satisfy this constraint, which is adopted especially for power allocation. The allocated power directly affects the signal-to-noise ratio at the receiver, and it correspondingly affects the secrecy sum-rates. Thus, the optimization of power allocation is significant for the optimization of secrecy sum-rates.

After the transmission of the uplink phase channel, the relay obtains the receiving signal as

(3) $$\begin{array}{cc} Y_{R}= & \sum_{i=1}^{N}H_{i,R}X_{i}+n_{R}\\ = & \sum_{i=1}^{N}H_{i,R}P_{i}c_{i}+n_{R}, \end{array}$$

where $H_{i,R}$ is the uplink channel state matrix. We assume that all channels are flat fading, so $H_{i,R}$ remains constant during the scheme with the entry of symmetric complex Gaussian random variables with a zero mean and unit variance. $n_{R}$ is the noise vector at the relay and modeled by $n_{r}∼CN\left(0,I_{N_{r}}\right)$. The estimation of $H_{i,R}$ will be discussed in the end of this subsection.

With the receipt of $Y_{R}$, the relay decodes and broadcasts the converged messages to all users, and the signal to be broadcast in the downlink phase is denoted by $X_{R}$. Similar to the uplink phase channels, the downlink phase channels are also assumed to be flat fading channels, and the channel matrix between the relay and user *i* is denoted by $G_{R,i}$. Thus, the received signal for user *i* in the downlink phase $Y_{i}$ can be written as

(4) $$Y_{i}=G_{R,i}X_{R}+n_{i},$$

where $n_{i}$ is the noise vector at user *i* and modeled by $n_{i}∼CN\left(0,I_{N_{i}}\right)$.

With the received broadcast signal from the relay, each user recovers the message transmitted to it with the help of its own message. The decoding at the user is detailed in a later section.

We will have a very brief discussion on the channel estimation for our proposed protocol. The introduced estimation algorithm is designed with the least overhead and computation cost, so the accuracy of this estimation is limited. Just like the other trade-off problem, we must choose either accuracy or low cost. More complicated channel estimation may be found in [42,43,44,45]; however, these approaches must be modified before they directly use in the multiuser relay channel.

It is also takes two time slots for the channel estimation for multiuser relay channel. In the first time slot, all of the user nodes send their training vector $s_{i}$ to the relay. The training vectors are from a orthonormal set, so $s_{i}\times s_{i}^{t}=I$ and $s_{i}\times s_{j}^{t}=0$.

By transmitting the training vector, the relay obtains the received signal as:

(5) $$Y_{R_{train}}=\sum_{i=1}^{N}H_{i,R}s_{i}+n_{R_{train}}.$$

In the second time slot, the relay broadcasts the received signal to all the users. Then, for user *j*, we have:

(6) $$\begin{array}{cc} Y_{j_{train}}= & G_{R,j}Y_{R_{train}}+n_{j_{train}}\\ = & G_{R,j}\sum_{i=1}^{N}H_{i,R}s_{i}+H_{R,j}n_{R_{train}}+n_{j_{train}}. \end{array}$$

Right multiplying $s_{j}^{t}$, and recalling $s_{i}\times s_{i}^{t}=I$ and $s_{i}\times s_{i}^{t}=0$, we have:

(7) $$\begin{array}{cc} {\tilde{Y}}_{j_{train}}= & Y_{j_{train}}s_{i}^{t}\\ = & G_{R,j}H_{j,R}+\tilde{n}, \end{array}$$

where $\tilde{n}=H_{R,j}n_{R_{train}}s_{j}^{t}+n_{j_{train}}s_{j}^{t}$, is the equivalent noise vector at the user *j*. In addition, we assume the up-link channels and down-link channels are reciprocal channels, then we have $G_{R,i}=H_{i,R}^{t}$. Thus, with (7), we can use maximum likelihood to decode and compute $H_{i,R}$.

### 2.2. Security Model

We propose a protocol that enables coinstantaneous private information exchange and public information exchange. These two types of information exchange face different potential security hazards.

Private Information Exchange

In the private information exchange, two of the multiple users exchange private information with the help of the relay. In this case, the attack can originate from three terminals: external eavesdroppers, untrusted relays, and other N−2 users.

External Eavesdroppers: Because of the broadcast nature of wireless communication, eavesdroppers exist in the network. External eavesdroppers try to recover private information from users by wiretapping the uplink phase channels as well as the downlink phase channels. In the uplink phase, we denote an eavesdropper that attacks user *j* by $E_{j}$. We assume that multiple eavesdroppers cannot collude. This assumption is significant and reasonable given that the transmitted signals from the users are independent. Therefore, all the eavesdroppers are also independent of each other. Moreover, if one eavesdropper can wiretap two users at the same time, this eavesdropper can be viewed as two separate eavesdroppers. The received signal at the eavesdropper contains two parts: the desired signal from the user that the eavesdropper is attacking and the equivalent noise signal from other users. The channel matrix between user *i* and eavesdropper *j* is $H_{E_{ij}}$. Therefore, the received signal at eavesdropper *j* is

(8) $$Y_{E_{j}}=\sum_{\begin{array}{c} i=1\\ i\neq j \end{array}}^{N}H_{E_{ij}}X_{i}+n_{E_{j}},$$

where $n_{E_{j}}$ is the noise modeled by $n_{E_{j}}∼CN\left(0,I_{N_{e}}\right)$.

Eavesdroppers can also wiretap the relay in the downlink phase; however, they cannot obtain more information because of the data processing inequality. Thus, we only consider uplink phase eavesdroppers in this work [46].

Untrusted Relay: In a pessimistic assumption, the relay itself may launch attacks on the information to be exchanged. With an untrusted relay, the relay itself first obeys the transmission protocol to accomplish the information exchange. However, the relay may also be curious about the transmitted message from the users. This type of relay is also known as an *honest-but-curious* relay. The relay has some obvious advantages in launching an attack in this scenario. First, the relay participates in the transmission and is an authenticated node. Thus, the authentication-based strategy does not stop this attack. Second, the relay is the receiver in the first phase of the scheme and the transmitter in the second phase, so the classical wiretap channel model is not suitable in such a situation. Finally, the relay has the authority to establish direct contact with messages from users according to the protocol.

Untrusted Users: In private information exchange, some users may also be curious about the private information transmitted by other users. Thus, an attack by untrusted users is considered in the private information exchange in addition to the above two types of typical attacks. In this situation, every user could be serving two roles: one is as an exchanger of private information, and the other is as an attacker to obtain other users’ private information. With the assumption that all nodes work according to the half-duplex model, users can only launch an attack after receiving the broadcast signal in the downlink phase, after which they try to recover other users’ private information.

Public Information Exchange: In public information exchange, a group information exchange is formed for all users. With this consideration, attacks can only originate from external eavesdroppers and untrusted relays. These two attacks are similar to those for private information, but, in this case, the attacks are curious about the group information rather than private information. Thus, we do not go detail about the attack model for public information exchange.

External Eavesdroppers: Public information eavesdroppers try to recover public information from users by wiretapping the uplink phase channels as well as the downlink phase channels. The assumption for such eavesdroppers is identical to that for eavesdroppers in private information exchange. In fact, the received signal at the eavesdropper contains both private information and public information. Thus, we do not distinguish between the private information eavesdropper and the public information eavesdropper.

Untrusted Relay: We also consider an untrusted relay in public information exchange to be an *honest-but-curious* relay. Thus, the relay tries to recover public information from the coding chain formed after the uplink transmission.

## 3. Proposed Protocol

In this section, a novel physical layer secure protocol that enables simultaneous private and public information exchange for a MIMO multiuser relay channel is proposed. As the core design principle, the signal alignment technique and physical layer network coding are conducted at each user node.

### 3.1. Negotiation Phase

Before the *real* transmission process, each node negotiates with the relay to confirm the transmission status and obtain the subchannel order number allocated to the user to exchange its private message and public message.

The negotiation phase contains three steps:

Step 1: the users who wish to exchange information send a request to the relay. For each user, the request also contains the number of the private information exchange partner. For example, if user *2* is willing to exchange information with users *3* and *5*, then user *2* will send number *3* and *5* inside the request.

Step 2: The relay collects the request from the users and computes the sorting of private information partners. Before sorting, the relay first matches the information partners. Notably, only the two users that send the request to the relay are allowed to exchange the private information. Users that fail to find a partner are fed back an invalid private information exchange sign with the partner number. The sorting rule is that a user with a small user number is sorted first. For two pairs of partners, the user numbers are first compared, and the smaller one is placed at the front of the sorting table. If the two pairs of partners have the same small user number (i.e., one user exchanging information with two different users), then we compare the other user’s number. After the sorting of all valid partners, a sorting table is formed.

Step 3: After sorting private information exchange partners, the relay feeds back information to the users. The information includes two parts: the sorting result for the private information partners and the information about the private data stream number. User *i* exchanges $d_{pri_{i}}$ private messages, and the total number of private information partners is denoted by $d_{pri}$. Clearly, we have $d_{pri}=2*d_{pri_{i}}$. We introduce how this information is used in detail in the section that describes the designing of the precoding matrix.

### 3.2. Uplink Phase

Similar to the description in the previous section, the message vector to be exchanged for user *i* is $m_{i}$. Each user applies physical layer network coding before transmission. In this work, we consider the simplest physical layer network coding, namely, Binary Phase Shift Keying (BPSK) coding. The encoding progress for user *i* can be written as

(9) $$c_{i}\left(j\right)=\left\{\begin{array}{cc} +1,\; & for\;m_{i}\left(j\right)=1,\\ -1,\; & for\;m_{i}\left(j\right)=0. \end{array}\right.$$

It is worth noting that this denotation does not distinguish between private information and public information. To distinguish these two types of information, we denote the message containing private information by $m_{i\_pri}$ and the message containing public information by $m_{i\_pub}$. Accordingly, the coded vectors are $c_{i\_pri}$ and $c_{i\_pub}$.

With the coded message, each user applies a precoding matrix to form the transmitting signal. The precoding matrix accomplishes three main functions: (1) it reduces the transmission interference, (2) aligns the private information of one exchange partner at the relay, and (3) forms a public information chain at the relay.

The three functions are carried out by designing the precoding matrix for user *i* as

(10) $$P_{i}=V_{i}\Sigma_{i}^{-1}U_{i}^{t}RL_{i}\Psi_{i}.$$

The precoding matrix contains four parts. The first part is the first three matrices that come from the Singular Value Decomposition (SVD) of the uplink channel matrix $H_{i,R}$ to reduce channel interference in the uplink channel. The second part is called rotation matrix R to improve the power loss of naive zero-forcing. The third part is called channel allocation matrix $L_{i}$, which allocates subchannels to intended users. The last part is the power allocation matrix, which allocates power to each subchannel.

With SVD, the uplink channel matrix $H_{i,R}$ can be rewritten as

(11) $$H_{i,R}=U_{i}\Sigma_{i}V_{i}^{t},$$

where $U_{i}$ and $V_{i}^{t}$ are orthogonal matrices defined by $U_{i}\cdot U_{i}^{t}=I$, $V_{i}^{t}\cdot V_{i}=I$ and the rows and columns of these two matrices are orthonormal. $\Sigma_{i}$ is a diagonal matrix that collects singular values of $H_{i,R}$, arranged in descending order.

With the first three matrices, $H_{i,R}\cdot P_{i}$ can be written as

(12) $$\begin{array}{cc} H_{i,R}\cdot P_{i}= & \underset{︸}{U_{i}\Sigma_{i}V_{i}^{t}\cdot V_{i}\Sigma_{i}^{-1}U_{i}^{t}}RL_{i}\Psi_{i}\\ \; & \;{\;}^{=I}\\ = & RL_{i}\Psi_{i}. \end{array}$$

It is known that the power loss is very serious with the zero-forcing approach; in particular, the channel matrix is ill-conditioned. Thus, it is desired to improve the naive zero-forcing approach to generate the subchannels. We review some literature which also concentrates on avoiding the power loss suffered with naive zero-forcing, especially the work in [47] is designed for MIMO relay channel. However, the work in that paper is designed for only two users, so we must modify the precoder there to satisfy the multiusers relay channel.

With zero-forcing for the physical layer network coding in MIMO relay channel, each user conducts channel inversion. Orthogonal subchannels are generated with zero-forcing precoder. To improve the power loss suffered by the naive zero-forcing, the work in [47] introduces a rotation by multiplying an orthogonal matrix for the eigendirections of the aligned subchannels. Because the rotation matrix is orthogonal, the orthogonality of the subchannels is preserved; however, the power loss is avoided. The detailed theoretical analysis for the benefit of such rotation can refer to [47].

Before designing the rotation matrix R, we first define a transition matrix T as:

(13) $$T≜\sum_{i=1}^{N}U_{i}\Sigma_{i}^{-2}U_{i}^{t}.$$

We take eigendecomposition to the transition matrix T as:

(14) $$T=U_{T}\Lambda_{T}U_{T}^{t},$$

where $U_{T}$ is an orthogonal matrix and $\Lambda_{T}$ is a diagonal matrix containing the eigenvalues of T.

With Equation (14), we choose the rotation matrix as:

(15) $$R=U_{T}.$$

Finally, we have some complement explanation that the computational of rotation matrix R is done by the relay, for only the relay is aware of all the channel states to compute the rotation matrix, and the relay is able to broadcast this matrix with least communication overhead.

The matrix $L_{i}$ is called the subchannel allocation matrix for user *i*. With this matrix, the user aligns its private information with its exchange partner(s) at the relay to form a superposition message. Simultaneously, the user aligns its public information with its two neighboring users to form a coding chain at the relay.

The design of the subchannel allocation matrix is relevant to the feedback from the relay in the negotiation phase. We take user *i* as an example. The design of the subchannel allocation matrix contains two parts: one is the design for private information $L_{i_{pri}}$, and the other is the design for public information $L_{i_{pub}}$. With the sorting table, user *i* computes the subchannel numerical order for the private information. Note that only the subchannels for user *1* are defined successively because of the sorting rule in the negotiation phase. We assume that user *i* exchanges private information with user *u* and user *v*. Pair (*i,u*) is the *m*th pair in the sorting table, and pair (*i,v*) is the *n*th pair. Then, $L_{i_{pri}}$ for user *i* is designed as

(16) $$L_{i_{pri}}^{t}=\left[\begin{array}{c} \begin{array}{c} \;\;m\\ \overset{︷}{000\cdots 0}10\cdots \;\cdots 00\\ \underset{︸}{000\cdots 0\cdots 0}10\cdots 0\\ \;\;n \end{array} \end{array}\right].$$

$L_{i_{pri}}$ has a size of $N_{r}*2$. In fact, the design $L_{i_{pri}}$ chooses the *m*th and *n*th columns of an $N_{r}*N_{r}$ identity matrix. With such a design, the message to be exchanged with *u* is allocated to the *m*th subchannel; similarly, the message to be exchanged with *v* is allocated to the *n*th subchannel.

The design principle for $L_{i_{pub}}$ is to allocate two successive subchannels to one user to form a coding chain. The subchannels for the public information exchange follow the subchannel of the previous private information exchange pair. The number of total private information pairs is $d_{pri}$, which is broadcast to the users in the negotiation phase. Thus, user *1* first aligns its message with user *2* in the $d_{pri}+1$th subchannel. Then, user *2* aligns its message with user *3* in the $d_{pri}+2$th subchannel. Similarly, each user from user *3* to user *N − 1* aligns its message with the previous and subsequent user. User *N − 1* aligns its message with user *N* in the $d_{pri}+N-1$th subchannel, but user *N* does not have a subsequent user. User *N* aligns its message with the first user in the $d_{pri}+N$th subchannel. Thus, user *1* is also allocated the $d_{pri}+N$th subchannel. We show the mathematical expression of $L_{i_{pub}}$ for i>1 in Equation (17):

(17) $$L_{i_{pub}}^{t}=\left[\begin{array}{c} \begin{array}{c} {\;}_{d_{pri}+i-1}\\ \overset{︷}{000\cdots 0}10\cdots 0\\ 000\cdots 001\cdots 0 \end{array} \end{array}\right].$$

For user *1*, $L_{1_{pub}}$ is designed as

(18) $$L_{1_{pub}}^{t}=\left[\begin{array}{c} \begin{array}{c} 100\cdots 00\\ 000\cdots 01 \end{array} \end{array}\right].$$

With $L_{i_{pri}}$ and $L_{i_{pub}}$ designed, user *i* computes the channel allocation matrix as

(19) $$L_{i}=\left[\begin{array}{c} L_{i_{pri}}\;L_{i_{pub}} \end{array}\right].$$

The last part of the design of the precoding matrix is the power allocation matrix $\Psi_{i}$. For user *i*, the power allocation vector allocates power to each subchannel created by the former matrices. The allocated power can linearly amplify the coded message to successfully reach the receiver (i.e., the relay). If more power is allocated to one subchannel, the transmitted signal enjoys a better chance of resisting error. However, the total power must usually satisfy the power constraint in Equation (2). Thus, designing the power allocation matrix is also a significant problem.

Usually, we use secrecy sum-rates as performance metrics for power allocation. The optimization of power allocation is significant for secrecy sum-rates, but the optimization problem under such a constraint is non-convex. One possible solution to this problem contains two steps. In the first step, each user applies a water-filling algorithm to its own subchannels, and, in the second step, the system finds the optimal solution to the secrecy sum-rates of the whole system. We mainly focus on proposing a practical protocol in this paper, so we do not go into detail about the optimization of power allocation. Furthermore, we only consider unitary power allocation in this paper, so all the elements in the power allocation matrix are the same. Thus, we take it as one in the rest of this paper for a better analysis. The optimization problem will be a significant part of future work that builds on this paper.

### 3.3. Relay Operation

After the precoding, all the users transmit their signals to the relay. With the precoding design in (10), the received signal at the relay can be rewritten as

(20) $$\begin{array}{cc} Y_{R}= & \sum_{i=1}^{N}H_{i,R}P_{i}c_{i}+n_{R}\\ = & \sum_{i=1}^{N}U_{i}\Sigma_{i}V_{i}^{t}V_{i}\Sigma_{i}^{-1}U_{i}^{t}RL_{i}c_{i}+n_{R}\\ = & R\sum_{i=1}^{N}L_{i}c_{i}+n_{R}. \end{array}$$

Due to the fact that R is an orthogonal matrix, we can obtain the equivalent received signal by left multiplying $R^{t}$. In addition, the subchannel allocation matrices are designed to align a user’s private information with its private information exchange partner and users’ public information with their two neighboring users. Then, the received signal at the relay can be rewritten as

(21) $$\begin{array}{cc} {\tilde{Y}}_{R}= & R^{t}Y_{R}\\ = & \left[\begin{array}{c} \begin{array}{cc} c_{1_{pri}}\left(1\right) & +c_{v_{pri}}\left(1\right)\\ c_{1_{pri}}\left(2\right) & +c_{u_{pri}}\left(1\right)\\ \; & \cdots \\ c_{N-1_{pri}}\left(l\right) & +c_{p_{pri}}\left(n\right)\\ c_{N-1_{pri}}\left(m\right) & +c_{q_{pri}}\left(j\right)\\ c_{1_{pub}} & +c_{2_{pub}}\\ c_{2_{pub}} & +c_{3_{pub}}\\ \; & \cdots \\ c_{N-1_{pub}} & +c_{N_{pub}}\\ c_{N_{pub}} & +c_{1_{pub}} \end{array} \end{array}\right]+n_{R}. \end{array}$$

From (21), we can clearly see that the received signal at the relay now is a summed signal plus the noise vector. In the next step, the relay removes the noise vector and decodes the summed signal back to the binary field. Recall that we assume that the noise vector is $n_{R}∼CN\left(0,I_{N_{R}}\right)$ and that the decoding rule is the threshold of the summed signal, so the decoded elements of the summed signal at the relay are

(22) $${\hat{Y}}_{sum}\left(i\right)=\left\{\begin{array}{cc} 1,\;\; & \left|Y_{R}\left(i\right)\right|\leq 1+\ln2/2,\\ 0,\;\; & \mathrm{otherwise}. \end{array}\right.$$

Note that ${\hat{Y}}_{sum}$ contains all private information as well as public information from all users. In addition, in view of the binary field, each bit of the decoded signal is a modulo-2 sum of two messages, that is, ${\hat{Y}}_{sum}\left(i\right)=m_{u_{pri}}⊕m_{v_{pri}}$ or ${\hat{Y}}_{sum}\left(i\right)=m_{i_{pub}}⊕m_{{i+1}_{pub}}$.

The relay applies BPSK encoding for ${\hat{Y}}_{sum}$ as

(23) $$c_{R}\left(j\right)=\left\{\begin{array}{cc} +1,\; & for\;{\hat{Y}}_{sum}\left(j\right)=1,\\ -1,\; & for\;{\hat{Y}}_{sum}\left(j\right)=0. \end{array}\right.$$

Then, the transmitting signal $X_{R}$ is generated for the downlink phase as $X_{R}=c_{R}\psi_{R}$, where $\psi_{R}$ is the power allocation vector for the downlink phase channels. Similar to the case for the uplink phase, we only consider unitary power allocation in this work.

### 3.4. Downlink Phase

The relay broadcast $X_{R}$ in the downlink phase is presented here. We take user *i* as an example. The main function of the downlink phase is that all the users correctly recover ${\hat{Y}}_{sum}$ and obtain the private and public information.

In contrast to the uplink phase, in which channel interference is reduced by the precoding, in the downlink phase, each user applies a detective matrix $D_{i}$ to carry out this function. Additionally, the detective matrix divides the messages transmitting to user *i*. For the received signal described in (4), we have ${\tilde{Y}}_{i}=D_{i}G_{R,i}X_{R}+n_{i}$. By designing $D_{i}=R^{t}U_{i}{\left(\Sigma_{i}^{t}\right)}^{-1}V_{i}^{t}$ and recalling that we have the reciprocal channel assumption defined by $G_{R,i}=H_{i,R}^{t}$, we have

(24) $$\begin{array}{cc} {\tilde{Y}}_{i}= & D_{i}H_{R,i}^{t}X_{R}+n_{i}\\ = & U_{i}{\left(\Sigma_{i}^{t}\right)}^{-1}V_{i}^{t}V_{i}\Sigma_{i}^{t}U_{i}^{t}X_{R}+n_{i}\\ = & c_{R}+n_{i}. \end{array}.$$

It is worth noting that ${\tilde{Y}}_{i}$ contains all the aligned private information as well as the public coding chain. User *i* obtains the aligned private information by left multiplying $L_{i_{pri}}$ as

(25) $$\begin{array}{cc} {\tilde{Y}}_{i_{pri}}= & L_{i_{pri}}*{\tilde{Y}}_{i}\\ = & \left[\begin{array}{c} \begin{array}{cc} c_{i_{pri}}\left(1\right) & +c_{u_{pri}}\left(1\right)\\ c_{i_{pri}}\left(2\right) & +c_{v_{pri}}\left(1\right)\\ \; & \cdots \\ c_{w_{pri}}\left(l\right) & +c_{i_{pri}}\left(d_{pri_{i}}-1\right)\\ c_{p_{pri}}\left(m\right) & +c_{i_{pri}}\left(d_{pri_{i}}\right) \end{array} \end{array}\right]+{\tilde{n}}_{i_{pri}}, \end{array}$$

where $\tilde{n_{i}}=L_{i_{pri}}*n_{i}$.

For (25), user *i* conducts decoding to recover the message from its private information exchange partner with the help of its own exchanged private messages. The decoding rule is

(26) $${\hat{m}}_{i_{pri}}\left(j\right)=\left\{\begin{array}{cc} 1⊕m_{i_{pri}}\left(j\right), & {\tilde{Y}}_{i_{pri}}\left(j\right)\geq 0,\\ 0⊕m_{i_{pri}}\left(j\right), & otherwise. \end{array}\right.$$

Thus, ${\hat{Y}}_{i_{pri}}\left(j\right)$ is the recovered exchanged private message from user *i*’s *j*th exchange partner.

For the public exchange information, user *i* obtains the coding chain and recovers all the other users’ public messages with the help of its own public message $m_{i_{pub}}$. Recall that the design places the public information coding chain in the $d_{pri}+1$th to $d_{pri}+N$th subchannels. Thus, we left multiply the matrix ${\hat{L}}_{i_{pub}}$ as

(27) $${\hat{L}}_{i_{pub}}=\left[0_{d_{pri}*d_{pri}}\;\;I_{N*N}\right].$$

With ${\hat{L}}_{i_{pub}}$, we extract the coding chain as

(28) $$\begin{array}{cc} {\tilde{Y}}_{i_{pub}}= & {\hat{L}}_{i_{pub}}*{\tilde{Y}}_{i}\\ = & \left[\begin{array}{c} \begin{array}{cc} c_{1_{pub}} & +c_{2_{pub}}\\ c_{2_{pub}} & +c_{3_{pub}}\\ \; & \cdots \\ c_{N-1_{pub}} & +c_{N_{pub}}\\ c_{N_{pub}} & +c_{1_{pub}} \end{array} \end{array}\right]+{\tilde{n}}_{i_{pub}}, \end{array}$$

where ${\tilde{n}}_{i_{pub}}={\hat{L}}_{i_{pub}}*n_{i}$.

With ${\tilde{Y}}_{i_{pub}}$, user *i* recovers the public message from all other users with successive decoding. First, user *i* decodes the messages from its two neighbors. Taking user *i − 1* as an example, we have

(29) $${\hat{m}}_{i-1_{pub}}=\left\{\begin{array}{cc} 1⊕m_{i_{pub}}, & {\tilde{Y}}_{i_{pub}}\left(i-1\right)\geq 0,\\ 1⊕m_{i_{pub}}, & otherwise. \end{array}\right.$$

We can similarly recover ${\hat{m}}_{i+1_{pub}}$.

With recovered ${\hat{m}}_{i-1_{pub}}$ and ${\hat{m}}_{i+1_{pub}}$, user *i* successively recovers ${\hat{m}}_{i-2_{pub}}$ and ${\hat{m}}_{i+2_{pub}}$, followed by ${\hat{m}}_{i-3_{pub}}$ and ${\hat{m}}_{i+3_{pub}}$ until ${\hat{m}}_{1_{pub}}$ and ${\hat{m}}_{N_{pub}}$. Thus, one round of information exchange is finished.

### 3.5. Example for N=3

We use an example to show how the precoding matrix provides the expected result. We set the user number to 3, and we assume that each user exchanges one private message and one public message with the other two users.

From Equation (10), we can see that the first three matrices are designed to reduce interference from the channel, and the power allocation matrix is a unitary matrix with unitary power allocation. Then, we can only focus on the design of $L_{i}$. According to the design principle, $L_{1}$ for user *1* is

(30) $$L_{1}=\left[\begin{array}{cccc} 1 & 0 & 0 & 0\\ 0 & 1 & 0 & 0\\ 0 & 0 & 0 & 0\\ 0 & 0 & 1 & 0\\ 0 & 0 & 0 & 0\\ 0 & 0 & 0 & 1 \end{array}\right].$$

With (30), the first, second, fourth, and last subchannels are allocated to user *1*. These four subchannels are used to transmit user *1*’s coded messages, and the detailed arrangement is that the first subchannel is used for aligning the private coded message $c_{1_{pri}}\left(1\right)$ with user *2*, the second subchannel is used for aligning the private coded message $c_{1_{pri}}\left(2\right)$ with user *3*, and the fourth and last subchannels are used for the public coded message $c_{1_{pub}}$. Thus, the code vector of user *1* is

(31) $$c_{1}=\left[\begin{array}{c} c_{1_{pri}}\left(1\right)\\ c_{1_{pri}}\left(2\right)\\ c_{1_{pub}}\\ c_{1_{pub}} \end{array}\right].$$

User *2* aligns $c_{2_{pri}}\left(1\right)$ with user *1* in the first subchannel, aligns $c_{1_{pri}}\left(2\right)$ with user *3* in the third subchannel, and aligns its public coded message $c_{2_{pub}}$ in the fourth and fifth subchannels. Thus,

(32) $$L_{1}=\left[\begin{array}{cccc} 1 & 0 & 0 & 0\\ 0 & 0 & 0 & 0\\ 0 & 1 & 0 & 0\\ 0 & 0 & 1 & 0\\ 0 & 0 & 0 & 1\\ 0 & 0 & 0 & 0 \end{array}\right].$$

Similarly, the design of $L_{1}$ is

(33) $$L_{1}=\left[\begin{array}{cccc} 0 & 0 & 0 & 0\\ 1 & 0 & 0 & 0\\ 0 & 1 & 0 & 0\\ 0 & 0 & 0 & 0\\ 0 & 0 & 1 & 0\\ 0 & 0 & 0 & 1 \end{array}\right].$$

We now discuss the received signal at the relay. Recalling Equation (20) and $H_{i,R}\cdot P_{i}=L_{i}\Psi_{i}$, we have

(34) $$Y_{R}=\left[\begin{array}{c} \begin{array}{cc} c_{1_{pri}}\left(1\right) & +c_{2_{pri}}\left(1\right)\\ c_{1_{pri}}\left(2\right) & +c_{3_{pri}}\left(1\right)\\ c_{2_{pri}}\left(2\right) & +c_{3_{pri}}\left(2\right)\\ c_{1_{pub}} & +c_{2_{pub}}\\ c_{2_{pub}} & +c_{3_{pub}}\\ c_{3_{pub}} & +c_{1_{pub}} \end{array} \end{array}\right]+n_{R}.$$

We can see that all the coded messages are all aligned in the desired subchannels. Thus, the uplink precoding matrix design can provide the expected result.

The relay broadcasts these aligned coded messages to the three users. After left multiplying the detective matrix for each user, the aligned coded messages can be obtained by the three users. According to Equations (25) and (28), each user extracts private aligned messages and the public coding chain. With user *1* as an example, the recovered private aligned messages are

(35) $$\begin{array}{cc} {\tilde{Y}}_{1_{pri}}= & L_{1_{pri}}*{\tilde{Y}}_{1}\\ = & \left[\begin{array}{c} \begin{array}{cc} c_{1_{pri}}\left(1\right) & +c_{2_{pri}}\left(1\right)\\ c_{1_{pri}}\left(2\right) & +c_{3_{pri}}\left(1\right) \end{array} \end{array}\right]+{\tilde{n}}_{1_{pri}}. \end{array}$$

With (35), user *1* conducts decoding and removes its own message following the rule in (26). Then, user *1* recovers the private messages $m_{2_{pri}}\left(1\right)$ and $m_{3_{pri}}\left(1\right)$ from users *2* and *3*.

Similarly, the recovered public coding chain is

(36) $$\begin{array}{cc} {\tilde{Y}}_{1_{pub}}= & {\hat{L}}_{1_{pub}}*{\tilde{Y}}_{1}\\ = & \left[\begin{array}{c} \begin{array}{cc} c_{1_{pub}} & +c_{2_{pub}}\\ c_{2_{pub}} & +c_{3_{pub}}\\ c_{3_{pub}} & +c_{1_{pub}} \end{array} \end{array}\right]+{\tilde{n}}_{i_{pub}}. \end{array}$$

With (36), user *1* recovers the public messages from the two other users following the decoding rule in (26). The process is identical for users *2* and *3*.

## 4. Performance Analysis

In this section, we present the performance analysis of the proposed information exchange protocol.

### 4.1. Transmission Performance Analysis

Because alignment is formed for *N* users at the relay, the total number of time slots is reduced in our proposed protocol. As described for protocol processing, the proposed protocol only costs two time slots. We denote the time slot cost as $TS_{PHY}$ for the proposed physical layer secure information exchange protocol, and $TS_{PHY}=2$. Note that $TS_{PHY}$ is a constant that does not change as the number of users increases.

To demonstrate the benefits of the time slot cost in our proposed protocol, we compare it with two well-known protocols in multiuser relay channels: the naive TDMA protocol and the network layer network coding protocol. For the naive TDMA protocol, only two users can participate in one round of information exchange. In each time slot, only one user can communicate with the relay in the half-duplex model; thus, four time slots are required in one round of information exchange. In total, $C_{N}^{2}*4$ time slots are required for all *N* users to carry out information exchange:

(37) $$TS_{TDMA}=C_{N}^{2}*4=2N*\left(N-1\right).$$

For naive TMDA, group information exchange is accomplished by arranging every two users’ exchange information in four time slots. However, this approach is extremely inefficient when the user number is large. The numerical results are shown in Section 5.

Network layer network coding is another approach to group information exchange for a multiuser relay channel. The network layer network coding approach can also be divided into two phases. In the first phase, each user sends its network layer network coded message to the relay successively in *N* time slots. In the second phase, the relay generates a converged message with all received coded messages and broadcasts it in the N+1th time slot to all users. Thus, the time slot cost of this protocol for *N* users is $TS_{Net}=N+1$.

Although the time slot cost still appears to be acceptable if the user number is not very large, only public information can be exchanged in an insecure approach. If an eavesdropper wiretaps the uplink channel, it can directly obtain transmitted messages. Moreover, private information is not available with the network layer network coding approach. These problems can all be solved by applying the proposed protocol, and, in the next subsection, we show how the proposed protocol protects users’ information from an external eavesdropper and an untrusted relay for both private and public messages.

### 4.2. Security Analysis

The proposed protocol is designed to enable two users to exchange private information and all users to exchange public information securely. As described in Section 2, for the private information exchange, we consider attacks from an external eavesdropper, an untrusted relay, and other users. For public information, we only consider attacks from an external eavesdropper and an untrusted relay.

#### Private Information Exchange

For private information exchange, three types of attacks should be considered. First, we should consider attacks from external eavesdroppers. To the external eavesdropper, the received signal is a superposed signal of each exchange pair. In contrast to the relay, which distinguishes these signals and orders them in their corresponding subchannels, the external eavesdropper receives every transmitted signal as artificial noise from all other signals. Thus, the external eavesdropper has difficulty recovering any exchanged information in the uplink phase. In the downlink phase, although the eavesdropper can directly wiretap the broadcasted signal, it can only obtain a summed signal that cannot be divided into the original individual information from the users.

With the untrusted relay, although the transmitted signals can be distinguished and ordered into the corresponding subchannels, the received signal in each subchannel is also an aligned signal of two users. Thus, the untrusted relay also cannot correctly recover the original individual information of each user.

For each exchange pair in the private information exchange, all other users can act as an attacker. The users can only receive signals in the downlink phase in the half-duplex model, so user *i* (acting as an attacker) can only launch an attack on the received $\tilde{Y_{i}}$. However, each user is only aware of its own arranged subchannel order, so it is difficult to obtain the particular subchannel number of a private information exchange pair. Furthermore, even if it can obtain a particular number of an exchange pair, the attacking user can only recover an aligned signal of two users in the same manner as the untrusted relay. Thus, we can conclude that the proposed protocol enables private information exchange in the multiuser relay channel.

#### Public Information Exchange

For public information exchange, two types of attacks should be considered: the external eavesdropper and the untrusted relay. Because each node can obtain public information, this information is not concealed from others. To the external eavesdropper, all the transmitted signals from users form a superposition of all eavesdroppers, and no eavesdropper can distinguish public information from each user.

To address an attack by the untrusted relay, we first recall that the public information forms a coding chain at the relay. The coding chain is from the $d_{pri}+1$th element to the $d_{pri}+N$th element of $Y_{R}$, and each element is the sum of two successful public information exchanges. Thus, the relay can only receive a summed signal that cannot be divided into the separated exchanged public information from each user.

#### Security Analysis of Some Other Well-Known Protocols

We also conducted a security analysis on some other well-known protocols for the multiuser relay channel. Because the TDMA protocol is limited in promoting security for exchanged information, we only discuss the cryptography approach, the network coding approach, and the secure group information exchange protocol proposed in [37].

In the cryptography approach, each node runs an encryption algorithm before the transmission. With encryption, attacks from external eavesdroppers can be strongly resisted. However, the existing encryption algorithms all need an authorized center to arrange the keys, and the relay always acts as the authorized center in group information exchange. Thus, this approach cannot resist the untrusted relay attack. Furthermore, the cryptography approach enables private information exchange and public information exchange with different encryption algorithms. Thus, this approach cannot allow simultaneous public and private information exchange. Besides these issues, the system and computation complexities are very high, so this protocol is not suitable for the power constraint of the system.

In the network coding approach, two phases are required to complete one round of information exchange. In the first phase, each node sends its coded information to the relay one by one, so it takes *n* time slots for *n* users. In the second phase, the relay broadcasts the converged coded information to all users. In the uplink phase, the eavesdropper can directly wiretap the transmitted information without any resistance, but the eavesdropper cannot recover the individual information from each user. Moreover, the relay receives the information from users one by one, so this approach cannot resist the untrusted relay. Thus, the network coding approach can only resist downlink eavesdroppers. Additionally, the network coding protocol is only designed for public information exchange.

The secure group information exchange protocol proposed in [37] is designed to resist both external eavesdroppers and untrusted relays. However, it can only enable public information exchange.

### 4.3. Secrecy Sum-Rate Analysis

In this section, we present the sum-rate analysis of the proposed protocol. Because the proposed protocol is designed to resist attacks from both untrusted relays and external eavesdroppers, the analysis must include three scenarios: a relay attack only, an eavesdropper attack only, and both a relay and eavesdropper attack.

#### Relay Attack Only

First, we consider the situation in which only an untrusted relay launches an attack. In this scenario, each user must regulate its secrecy transmission rate to guarantee that the relay cannot correctly recover the information being exchanged. Thus, the general secrecy sum-rate expression is

(38) $$R_{s_{UR}}=\frac{1}{2}{\left[\sum_{i=1}^{N}R_{i_{UR}}-R_{R_{UR}}\right]}^{+},$$

where $R_{i_{UR}}$ is the transmission rate of user *i* for such attack and is defined by

(39) $$R_{i_{UR}}=\log\;\det\left(I+H_{i}Q_{i}H_{i}^{t}\right).$$

$R_{R_{UR}}$ is the information received at the relay from the transmitted signals from all users. Thus, $R_{R_{UR}}$ is

(40) $$\begin{array}{cc} R_{R_{UR}}= & I\left(Y_{R};X_{1},X_{2}\cdots X_{N}\right)\\ = & \log\;\det\left(I+\sum_{i=1}^{N}H_{i}Q_{i}H_{i}^{t}\right). \end{array}$$

With (39) and (40), (38) can be rewritten as

(41) $$\begin{array}{cc} R_{s_{UR}}= & \frac{1}{2}{\left[\sum_{i=1}^{N}R_{i_{UR}}-R_{R_{UR}}\right]}^{+}\\ = & \frac{1}{2}\log\;\det\left[\frac{\prod_{i=1}^{N}\left(I+H_{i}Q_{i}H_{i}^{t}\right)}{I+\sum_{i=1}^{N}H_{i}Q_{i}H_{i}^{t}}\right]. \end{array}$$

#### Eavesdropper Attack Only

Second, we consider the situation in which the relay is a trusted node, but there exists an eavesdropper between the users and relay. We limit our considerations to the case in which only one eavesdropper exists between each user and the relay. In a practical situation, the number of eavesdroppers may be larger than one, but we only consider the eavesdropper that obtains the largest $I\left(Y_{E_{j}};X_{i}\right)$. If the protocol is secure from an attack in which an eavesdropper obtains the most information from the user, then the protocol is definitely secure from all other eavesdropper attacks from the perspective of information-theoretic security.

As mentioned above, the eavesdropper cannot launch a strong attack in the downlink phase because of the data processing inequations, so we also consider only an uplink phase eavesdropper. In the uplink phase, each user forms a typical wiretap channel with the relay and its eavesdropper. Thus, the sum rates for this attack is the sum of all *N* wiretap secrecy rates:

(42) $$R_{s_{EA}}=\frac{1}{2}{\left[\sum_{i=1}^{N}\left(R_{i_{EA}}-R_{{Ei}_{EA}}\right)\right]}^{+},$$

where $R_{i_{EA}}$ is the transmission rate of user *i* for an eavesdropper-only attack, and its analysis is identical to that of (39):

(43) $$R_{i_{EA}}=\log\;\det\left(I+H_{i}Q_{i}H_{i}^{t}\right).$$

$R_{{Ei}_{EA}}$ is the information obtained by eavesdropper *i*. We use the variable $\nu_{i}$ to represent the existence of eavesdropper *i*: $\nu_{i}=1$ if there exists an eavesdropper between user *i* and the relay; otherwise, $\nu_{i}=0$.

(44) $$\begin{array}{cc} R_{Ei_{EA}}= & I\left(Y_{E_{i}};X_{i}\right)\\ = & \log\;\det\left(I+\nu H_{E_{ii}}Q_{i}H_{E_{ii}}^{t}\right). \end{array}$$

With (43) and (44), (42) can be written as

(45) $$\begin{array}{cc} R_{s_{EA}}= & \frac{1}{2}{\left[\sum_{i=1}^{N}\left(R_{i_{EA}}-R_{R_{EA}}\right)\right]}^{+}\\ = & \frac{1}{2}\log\;\det\left[\prod_{i=1}^{N}\left(\frac{I+H_{i}Q_{i}H_{i}^{t}}{I+\nu H_{E_{ii}}Q_{i}H_{E_{ii}}^{t}}\right)\right]. \end{array}$$

#### Untrusted Relay and Eavesdropper Attack

Finally, we consider the undesirable situation in which the relay is an untrusted relay and there also exist eavesdroppers between the user and the relay. The secrecy sum-rate analysis is a combination of the first two situations:

(46) $$R_{s_{UE}}=\frac{1}{2}{\left[\sum_{i=1}^{N}\left(R_{i_{UE}}-R_{Ei_{UE}}\right)-R_{R_{UE}}\right]}^{+}.$$

The analyses of $R_{i_{UE}}$, $R_{Ei_{EA}}$, and $R_{Ei_{EA}}$ are identical to those of the previous two situations:

(47) $$R_{i_{UE}}=\log\;\det\left(I+H_{i}Q_{i}H_{i}^{t}\right),$$

and

(48) $$\begin{array}{cc} R_{Ei_{UE}}= & I\left(Y_{E_{i}};X_{i}\right)\\ = & \log\;\det\left(I+\nu H_{E_{ii}}Q_{i}H_{E_{ii}}^{t}\right), \end{array}$$

and

(49) $$\begin{array}{cc} R_{R_{UE}}= & I\left(Y_{R};X_{1},X_{2}\cdots X_{N}\right)\\ = & \log\;\det\left(I+\sum_{i=1}^{N}H_{i}Q_{i}H_{i}^{t}\right). \end{array}$$

With (47), (48), and (49), (46) can be written as

(50) $$\begin{array}{cc} R_{s_{UE}}= & \frac{1}{2}{\left[\sum_{i=1}^{N}\left(R_{i_{UE}}-R_{Ei_{UE}}\right)-R_{R_{UE}}\right]}^{+}\\ = & \frac{1}{2}\log\;\det\left[\frac{\prod_{i=1}^{N}\left(\frac{I+H_{i}Q_{i}H_{i}^{t}}{I+\nu H_{E_{ii}}Q_{i}H_{E_{ii}}^{t}}\right)}{I+\sum_{i=1}^{N}H_{i}Q_{i}H_{i}^{t}}\right]. \end{array}$$

## 5. Numerical Results

In this section, we show the numerical results for the time slot cost and the secrecy sum-rate analysis of the proposed protocol. The simulation environment was set up with the following conditions. (1) The user number is 4. Each user node is equipped with four antennas, the relay is equipped with eight antennas, and each eavesdropper is equipped with four antennas. (2) BPSK physical layer network coding is applied as the design. (3) With the assumption of a flat fading channel, the coefficients of channel matrices are drawn from CN(0,1). (4) The results were obtained with 10,000 realizations by randomly generating 10,000 channels for each simulation.

### 5.1. The Simulation of Time Slot Cost

For the time slot cost, we compared the proposed protocol with the TDMA protocol and network layer network coding.

**Figure 2:**
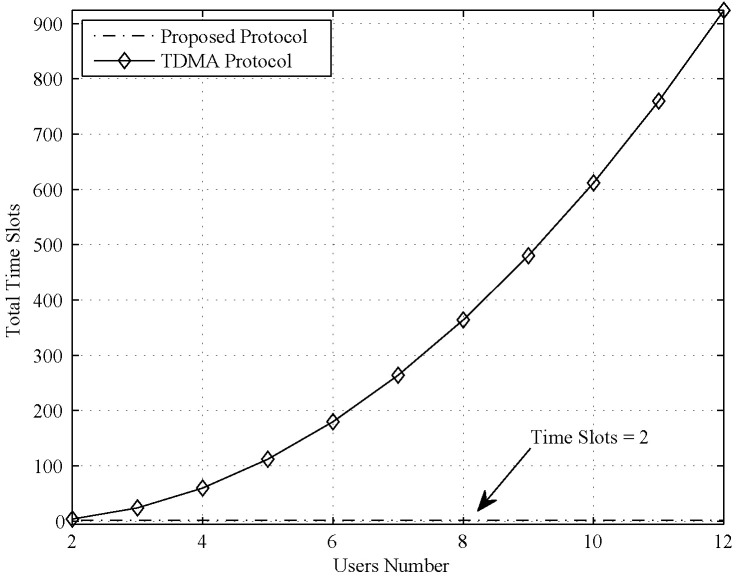
Time slot cost comparison between the proposed protocol and the naive TMDA protocol.

**Figure 3:**
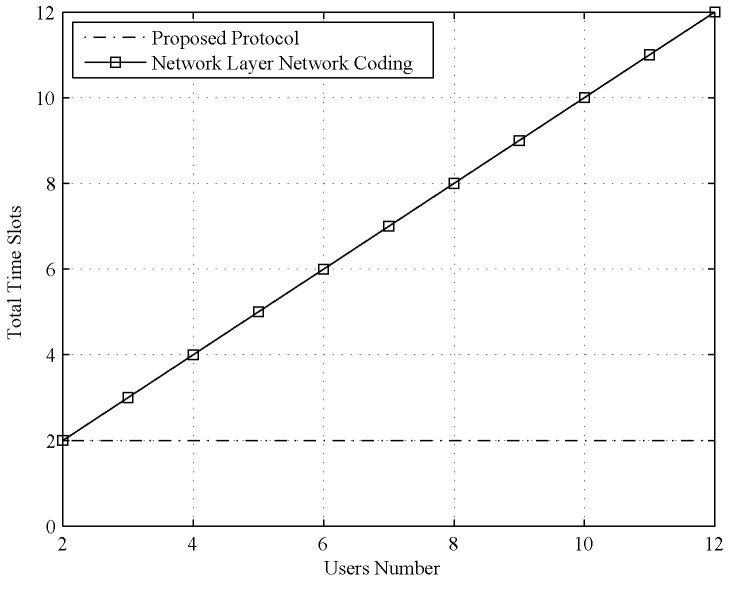
Time slot cost comparison between the proposed protocol and the network layer network coding protocol.

First, we compared the time slot cost of the proposed protocol with that of the naive TDMA protocol, and the comparison result is shown in Figure 2. As clearly seen in Figure 2, the time slot cost is larger than 100 when the user number is larger than 5. Moreover, for naive TMDA, users cannot exchange private information because the transmitted signal cannot find an equivalent noise signal in each time slot. The comparison result of the proposed protocol and network layer network coding is shown in Figure 3.

Second, we compared the time slot cost of the proposed protocol with that of the network layer network coding protocol, and the result is shown in Figure 3. The comparison result reveals that the time slot cost of the network coding protocol grows linearly with an increasing number of users. Although the cost of network coding is lower than that of the naive TDMA protocol, it is still several times larger than that of the proposed protocol. This benefit is pronounced when the user number is large.

### 5.2. The Simulation of the Bit Error Ratio

In the next simulation, we compared the bit error ratio (BER). We choose three types of nodes to compare, user node, relay node, and eavesdropper node. In the simulation, for all three of these types of nodes, we set the noise as 1 and adopt the transmission power to achieve the fixed SNR value and then obtain the BER.

Separately saying, for the user node, all the users only receive signal in the second phase. In each round, we randomly choose one user from the total *N* user nodes to compute the BER. For the SNR, according to the aforementioned, we set the noise at each antenna as 1, then the noise vector is an all 1 $N_{i}\times 1$ vector. In addition, we compute back the signal power for the relay and allocate the power to the relay. Such situation is all the same for the users including the chosen one. With each SNR, we obtain the error number and record it. After 10,000 rounds, we take the average BER as the final result for user node.

For the relay node, it only receives the signal in the first phase of the protocol. Because we have assumed that we only consider unitary power allocation in this work, we allocate the same power for each user. In addition, the allocated power will increase with the increasing of SNR. The BER of relay node is also obtained by 10,000 rounds.

**Figure 4:**
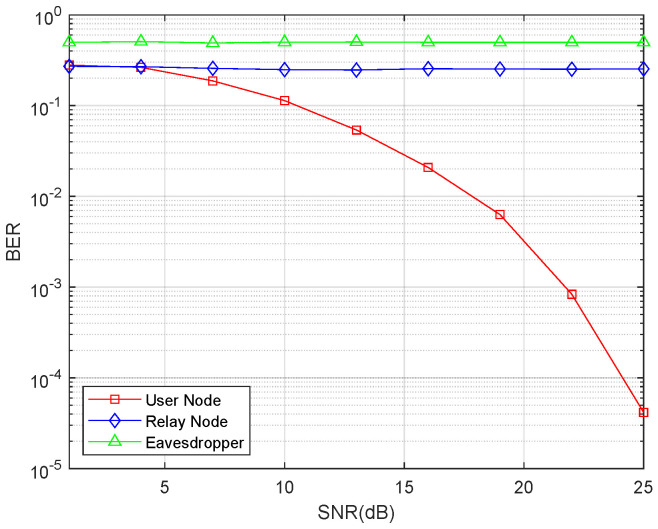
Bit error ratio comparison between the intended receive node, relay node, and eavesdropper node.

For the eavesdropper node, it tries to recover the transmitted data in both phases. The first phase is similar to the situation of the relay node, and the second phase is similar to the situation of the user node. The result of the BER comparison is shown in Figure 4.

Figure 4 illustrates that the BER markedly decreases as the Signal-to-Noise Ratio (SNR) increases. However, the BERs of the untrusted relay and eavesdropper remain high irrespective of changes in SNR. This result reveals that the information exchange would be successful with the proposed protocol, and the untrusted relay and external eavesdropper cannot recover the information.

### 5.3. The Simulation of Secrecy Sum-Rates

**Figure 5:**
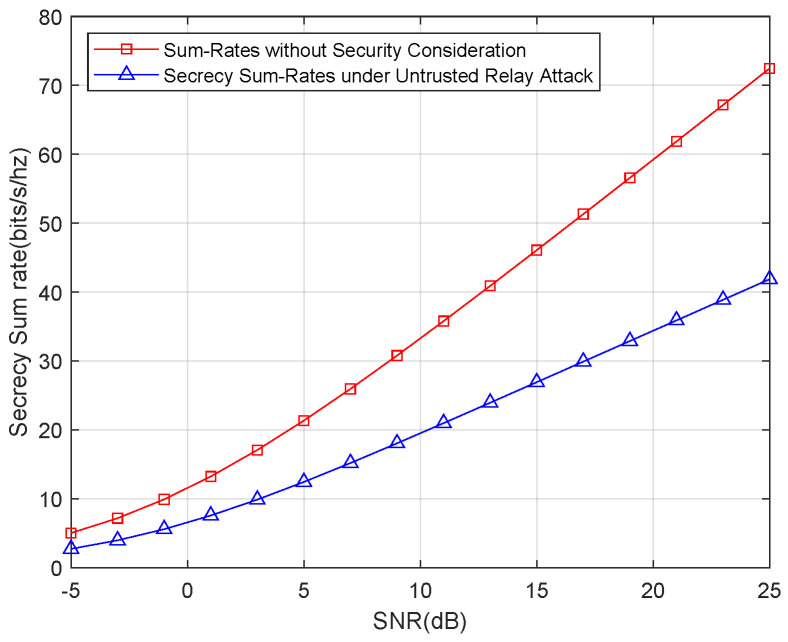
Secrecy sum-rates for an untrusted relay attack.

First, we show the secrecy sum-rates for only an untrusted relay attack. The result of the simulation is shown in Figure 5. For a better illustration, we also show the sum rates without the security consideration. Figure 5 shows that the secrecy sum-rates suffer a remarkable decline compared with the sum rates without the security consideration. This result highlights the trade-off between security and transmission efficiency; in other words, transmission rates are sacrificed to increase the security level.

**Figure 6:**
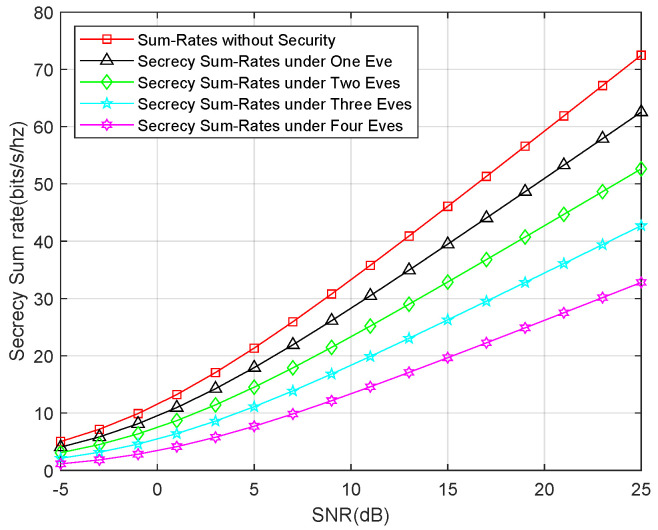
Secrecy sum-rates for the external eavesdropper attack.

Second, we show the secrecy sum-rates for an external eavesdropper attack. We compared the sum rates for one eavesdropper, two eavesdroppers, three eavesdroppers, and four eavesdroppers, as well as the sum rates without the security consideration. The result is depicted in Figure 6, which shows that the secrecy sum-rates linearly decrease as the number of eavesdroppers increase. Interestingly, the secrecy sum-rates for only the untrusted relay attack are approximately equal to the secrecy sum-rates for three eavesdroppers. This demonstrates that the untrusted relay can launch a stronger attack than an individual eavesdropper. This result is in accordance with the theoretical analysis.

**Figure 7:**
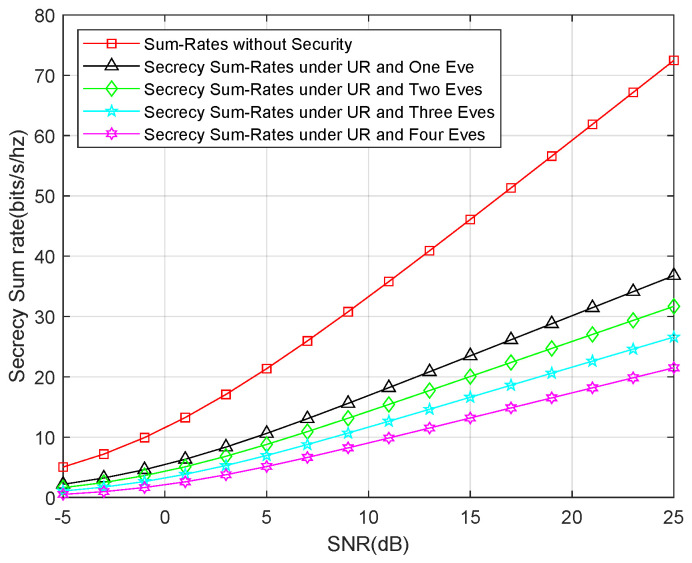
Secrecy sum-rates for an untrusted relay and an external eavesdropper attack.

Finally, we show the secrecy sum-rates for both an untrusted relay and external eavesdropper attack. Similar to the last simulation, we also compared the sum rates for one eavesdropper, two eavesdroppers, three eavesdroppers, and four eavesdroppers, as well as the sum rates without the security consideration. The result is shown in Figure 7, which shows that the secrecy sum-rates decline to one-third of the sum rates without the security consideration. This means that the security consideration is more important for this type of attack.

### 5.4. Comparisons with Well-Known Protocols

We compared our proposed protocol with some of the other four approaches in multiusers relay channel, i.e., cryptography approach, TMDA approach, network layer coding approach, and a physical layer network coding approach. The result has been shown in Table 1.

For the attackers, the proposed protocol can resist the attacks from both the eavesdroppers and untrusted relay. The cryptography approach can resist both attacks with the encryption. In addition, the physical layer network coding can resist both attacks by signal alignment similar to the proposed protocol. The network layer coding can only resist the downlink eavesdropper because the summed signal is formed only in that phase. The TMDA approach is only designed for plain data exchange, and no security issues are considered in such approach.

Recalling the motivation of this work, we want to design the first protocol that can simultaneously exchange private data and public data. For the cryptography approach, there are existing group encryption algorithms for either private or public exchange. However, none of the algorithms are able to exchange simultaneously. Both the network layer coding and physical layer coding can exchange public data; however, neither of them can exchange private data.

The overhead is also important for the communication protocol. Because the cryptography approach needs all nodes to conduct encryption and decryption, the computation overhead for such approach is very high. For the other four approaches, each node only does some linear computation or simple encoding and decoding, so the computation is low. For the communication overhead, the time slot cost is the best indicator. With the simulation result in Figure 2 and Figure 3, we can clearly see that the communication overhead of TDMA and cryptography approach (equal to TDMA) is high; in particular, the user number is large. The communication overhead of network layer coding is a little higher than the proposed protocol or physical layer network coding, but it is still acceptable.

With the comparisons, we can clearly see that our proposed protocol is the first and only protocol enabling the simultaneous information exchange for both private exchange and public exchange, which satisfies the motivations of the paper. However, there are two drawbacks for the proposed protocol. The first one is that the cryptography approach, TDMA approach, and network layer coding have been introduced into communication for many years. Thus, it will take more money to deploy our protocol. The second drawback is that our protocol is designed particularly for MIMO communication. Thus, for the SISO, SIMO, or MISO situations, only a cryptography approach can be used for either private information or public information. However, the good news is that more and more devices, even the delicate mobile phones, are equipped with multiple antennas.

## 6. Conclusions and Future Work

In this paper, we investigate secure private and public information exchange in a multiuser relay channel. The private information of each communication pair is aligned by allocating the information from each user into specified subchannels, and the public information of all users form a coding chain at the relay. The time slot cost of the proposed protocol is 2, which is a minimized number in the half-duplex model. A security analysis was conducted, and we show that both private and public information can resist attacks from external eavesdroppers and an untrusted relay, and private information can additionally resist an attack by nonparticipating users. We analyzed the secrecy sum-rates of the proposed protocol for three different attacks: an untrusted relay attack only, an external eavesdropper attack only, and both an untrusted relay and external eavesdropper attack. The performance analysis results are demonstrated by the outcomes of the simulations. The proposed protocol is the first approach enabling the simultaneous information exchange for both private exchange and public exchange. In addition, compared with the well-known approaches, the proposed protocol enjoys better performance in transmission efficiency as well as information security.

Future work that will build on this paper includes the optimization of the secrecy sum-rates, the asynchronous problem for signal alignment, and the application of the protocol to group secret key agreement. Another future work that should be conducted is related to practicability. With such motivation, we are trying to deploy our proposed protocol on a software defined radio experiment platform.

## Figures and Tables

**Table 1:** Comparisons of multiuser relay channel information exchange protocols.

Scheme	Proposed	Cryptography	TMDA	NetCoding	PHY Coding
Eavesdropping Resist	Yes	Yes	No	Downlink	Yes
Untrusted Relay Resist	Yes	Yes	No	No	Yes
Public Exchange	Yes	Yes	No	Yes	Yes
Private Exchange	Yes	Yes	No	No	No
Simultaneous Exchange	Yes	No	No	No	No
Computation Overhead	Low	High	Low	Low	Low
Communication Overhead	Low	High	High	Medium	Low
